# Factors associated with place of delivery in rural Nepal

**DOI:** 10.1186/1471-2458-14-306

**Published:** 2014-04-03

**Authors:** Sudesh Raj Sharma, Amod Kumar Poudyal, Bharat Mani Devkota, Sarswoti Singh

**Affiliations:** 1 The University of Sydney, Sydney, Australia; 2 Department of Community Medicine and Public Health, Maharajgunj Medical Campus, Tribhuvan University, Kathmandu, Nepal; 3 Equal Access International, Pulchowk, Kathmandu, Nepal

**Keywords:** Institutional delivery, Wealth quintile, Women’s autonomy, Adverse pregnancy outcome

## Abstract

**Background:**

Promotion of institutional delivery is a key intervention in reducing maternal mortality and improving maternal and neonatal health. This study explored factors associated with institutional delivery in rural Nepal.

**Method:**

A household survey was conducted in three rural Village Development Committees of Kavrepalanchowk district to identify the individual, household and health service factors associated with the institutional delivery. All 240 eligible mothers from the study area were interviewed during the study period. Multiple logistic regression analysis was applied to establish the factor associated with the institutional delivery, the outcome variable.

**Results:**

Antenatal care practice, adverse pregnancy outcome, ethnicity and time taken to reach the health institution were significantly associated with the institutional delivery. Utilization of an antenatal care service had the greatest effect on institutional delivery.

**Conclusion:**

Universal antenatal care service utilization may be a critical intervention for increasing institutional delivery. There is a need to raise awareness in hard-to-reach areas where adverse pregnancy outcomes is not considered a serious event.

## Background

Institutional delivery is an essential intervention in saving the lives of mothers and children in many developing countries. It ensures comprehensive medical attention and aseptic conditions during delivery, reducing the risk of complications and infections [1]. In Nepal, the practice of giving birth at home remains high, especially in rural areas [2]. As a result, Nepal’s efforts at reducing maternal mortality rate to 134 deaths per 100,000 live births, as per the *Millennium Development Goals* and the *Nepal Safe Motherhood and Neonatal Health Long Term Plan 2006–2017,* remains a challenge [3]. The government of Nepal is nonetheless making an effort to increase the utilization of maternal health services [4]. However, service delivery is currently ineffective due to limited information about determinants regarding service utilization by communities and individuals, particularly in rural areas. Multiple studies suggest the choice of place for childbirth is influenced by social, demographic, economic, geographic and health service factors [2,4-11]. Hence, the objective of this research was to identify the factors associated with institutional delivery among mothers with children below one year of age in the three Village Development Committees (VDCs) of Kavrepalanchowk district.

## Method

### Study setting

The study was conducted in Kavrepalanchowk district of the Central Development Region of Nepal. The Kavrepalanchowk district has a population of 385,672 with 86.3% of population residing in rural areas [12]. In order to represent the rural setting of Kavrepalanchowk district, three VDCs were purposefully selected based on their distance from the district headquarter. Meche VDC was the farthest of all, Chatrebanjh VDC was second closest, and Patlekhet VDC was the nearest from the district headquarter (68 kilometers, 23 kilometers and 10 kilometers respectively) [12]. District Health Office, Kavrepalanchowk provided necessary support to conduct the study in coordination with its sub-ordinate health institutions.

### Ethical statement

Ethical approval was obtained from the Ethical Review Board of Maharajgunj Medical Campus. The purpose and voluntary nature of the study was explained to participants prior to conducting the interview. Written consent was taken either from the participant or their guardian. Participants were interviewed by enumerators specially oriented on ethical issues associated with the study. Participant’s right to refuse to answer any question or discontinue interview at anytime was fully respected and adhered to.

### Study design and sampling

The study design was cross-sectional and descriptive. Mothers, who had delivered their child between 15 July 2010 and 14 July 2011 within the selected study area, were the participants in the survey. The survey was conducted over six months, from July 15 to December 31 2011. All 240 eligible mothers available at three VDCs during the survey period were selected for structured interview.

### Data collection procedure

Face to face interview was conducted to record information related to possible factors associated with place of giving birth using an adapted questionnaire. The questionnaire was adapted from the Nepal Demographic Health Survey, end-line survey of Equity and Access Program and a similar study in Pakistan [2,5,13]. The questionnaire was initially prepared in English and translated into Nepali. The Nepali version was pretested amongst mothers in Paanchkhal VDC. Three trained enumerators were mobilized for data collection. They were apt in local language and had higher secondary level of education.

### Measures

#### Outcome variables

The outcome variable of the study was institutional delivery. Institutional delivery was defined as the practice of delivery/giving birth by a mother in a health institution between 15 July, 2010 and 14 July, 2011. The outcome variable was validated by asking the mother for the name of the health institution she visited and about the type of health workers who assisted her during delivery.

#### Explanatory variables

The explanatory variables were studied under three categories: individual, household and health service factors. The roles of individual factors, which included mother’s current age, parity, past experience of adverse pregnancy outcome, literacy, occupation and antenatal care practice, were assessed. Literacy and occupational status of the study participants’ husbands’ was also assessed. The age of the respondent as reported by the individuals was recorded. For analysis purposes, age was categorized into four groups (less than 19, 20–24, 25–30 and above 30 years). Parity was classified as primiparous (mothers who had delivered only one child) and multiparous (mothers who had delivered more than one child).

Literacy status was identified by asking mothers whether they had attended formal school, and if they had, then the total number of years of formal schooling was recorded and the respondent was later categorised into one of the four levels: primary, secondary, intermediate, or bachelor and above. If the mother did not go to school, she was requested to read a card with a short statement on nutrition in order to identify whether she was just literate to a degree or illiterate entirely. During descriptive analysis, six levels of education were recognised: illiterate, just literate, primary, secondary, intermediate, bachelor and above. However, for bivariate analysis, these levels were reduced to two categories: illiterate and literate. The literacy status of the respondent’s husband was measured based on the response of the mother. Occupation was broadly classified into four categories: agriculture, labour, business and service. During bivariate analysis, occupation was regrouped into labour-intensive occupations (agriculture and labour) and non labour–intensive occupations (service/business). Experience of adverse pregnancy outcome was assessed by asking mothers whether they had experienced stillbirths or abortion during their lifetime. Mothers were requested to recall the frequency of antenatal care visits during last pregnancy, along with the services they received. The mothers were then categorized as either having had less than four visits, and those who had four or more visits.

In household factors, the association of ethnicity, socio-economic status (wealth quintile), women’s autonomy, and family type were examined. The Health Management Information System section of the Department of Health Services of Nepal has classified ethnicity into the following categories: Dalit, disadvantaged Janjati (indigenous people), relatively advantaged Janjati, other excluded groups including Terai based ethnic group (except Terai Brahmins), religious minorities and Brahmin/Chhetri. During multivariate analysis, the ethnic groups were classified into two groups: privileged and underprivileged ethnic groups. Brahmin/Chhetri and relatively advantaged Janjati (Newar, Thakali and Gurung) were classified as privileged ethnic groups, while Dalit, disadvantaged Janjati, Terai caste (excluding Terai Brahmins) and religious minorities were classified as underprivileged ethnic group; which was based on economic and educational differences between the two groups [14]. Socio-economic status was ascertained by measuring each group’s placement over the wealth quintile (used in the *Demographic Health Survey*), which involved principle component analysis (PCA) of details of household assets and expenditures [15]. Households were categorised into five ranked groups; the first rank representing the poor and the fifth rank representing the least poor. Women’s autonomy was measured by the help of 12 variables reflecting autonomy areas such as control over finances, decision-making power and the extent to which women have freedom of movement; adapted from a study in Pakistan [5]. Using PCA techniques, households were categorised into three ranked group. The first rank represented low autonomy group to third rank representing high autonomy group.

At the health institution level, the association of time taken to reach health institution, exposure to maternal health messages during pregnancy, and perceived behaviour of service provider during antenatal care with institutional delivery was assessed. For analysis purposes, the time taken to reach the health institution was divided into two categories: less than 60 minutes and more than 60 minutes, based on a study in Nepal [11]. Exposure to message was defined as the exposure of the respondents to messages relating to maternal health and service utilization during pregnancy through mass media and health workers. Mothers were requested to recall and rate the behaviour of health workers during their first antenatal care visit. The behaviour of health workers was classified as favourable or unfavourable, as perceived by the respondents.

### Analysis

Data compilation, checking, editing and coding was carried out by the researcher each day for quality assurance. The data from the questionnaire-based survey was entered in Epi Info™ 3.5.2 (CDC, USA), based on the codes provided to the questions and their entries. The analysis was conducted using SPSS 17 (IBM SPSS Inc., USA). Descriptive analysis was performed as per the study variables at the initial stage. In bivariate analysis, all variables significant at 0.1 level of significance were considered for multivariate analysis. Multivariate logistics regression analysis was conducted, at 0.05 level of significance, to identify factors associated with institutional delivery. The Hosmer–Lemeshow chi-square test was used to test the goodness of fit for the regression model [16].

## Results

### Description of delivery service utilization

Institutional delivery was very high in Kavrepalanchowk district compared to the national average of 35.3% for institutional delivery [2]. Proportion of institutional delivery in Kavrepalanchowk district was 65.5%.

### Description of individual, household and health service factors

Table 1 provides description of the individual factors. Nearly half of the mothers (49%) were from age group 20–24 and one in three (34%) from age group 25–30. About 44% of the mothers were primiparous and 28.8% had two children. Thirty percent of mothers and 15.4% of husbands were illiterate. Four in five mothers and 50.8% of husbands were engaged in agriculture. Proportions of mothers who had experienced adverse pregnancy outcome were 18.3%, and who had four or more antenatal care visits were almost 80%.

**Table 1:** Description of individual factors

**Characteristics**	**Number (n = 240)**	**Percentage**
Current age in years		
Less than 20	12	5.0
20-24	117	48.8
25-29	82	34.2
30 and above	29	12.1
Parity		
1	106	44.2
2	69	28.8
3	39	16.3
4	14	5.8
5 or more	12	5.0
Education (mother)		
Illiterate	72	30.0
Literate only	10	4.2
Primary	68	28.3
Secondary	65	27.1
Certificate level	19	7.9
Bachelors and above	6	2.5
Education (husband)		
Illiterate	37	15.4
Literate	3	1.3
Primary	17	7.1
Secondary	137	57.1
Certificate level	36	15.0
Bachelors and above	10	4.2
Occupation (mother)		
Agriculture	180	75.0
Business	11	4.6
Service	11	4.6
Labour	3	1.3
House work	35	14.6
Occupation (husband)		
Agriculture	122	50.8
Business	41	17.1
Service	38	15.8
Labour	31	12.9
House work	8	3.3
Experience of adverse pregnancy outcome		
No	196	81.6
Yes	44	18.3

Table 2 provides a description of the household and health service factors. Just over 70% of the mothers were from the underprivileged (Tamang and Dalit) ethnic group. The ranked groups within women’s autonomy and wealth quintile consisted of equal proportion of respondents (33.3% and 20% respectively). Nearly 68% of the mothers were from joint families while 32% belonged to nuclear families. Just over 80% of the mothers lived less than 60 minutes away from a health institution with birthing facilities. Forty percent of the mothers had heard of maternal health promotion information during pregnancy and 51.2% rated the behaviour of health worker during antenatal care check-up as favourable.

**Table 2:** Description of household and health service factors

**Characteristics (household factors)**		
Ethnicity		
Brahmin/Chhetri	62	25.8
Tamang	151	62.9
Newar	9	3.8
Dalit	18	7.5
Women’s autonomy		
Low	80	33.3
Medium	80	33.3
High	80	33.3
Wealth quintile		
First (poor)	48	20.0
2	48	20.0
3	48	20.0
4	48	20.0
Fifth (least poor)	48	20.0
Family type		
Nuclear	77	32.1
Joint	163	67.9
Characteristics (health service factors)		
Time taken to reach the health institution		
Less than 60 minutes	194	80.8
More than 60 minutes	46	19.2
Exposure to information related to maternal within during pregnancy		
Yes	96	40.0
No	144	60.0
Behaviour of health workers during antenatal care*		
Favourable	113	51.2
Unfavourable	108	48.8

*19 respondents were excluded during analysis as they did not go for antenatal care service utilization and hence did not experience the behavior of health workers during antenatal care.

### Factors associated with delivery service utilization

The association between institutional delivery and possible individual, household and health service factors was assessed at 95% CI in the multivariate analysis (Table 3). The factors that were significant during bivariate analysis were parity (p-value 0.036), occupation of mother (p-value 0.09) and husband (p-value <0.001), literacy status of mother (p-value <0.001) and husband (p-value 0.02), experience of adverse pregnancy outcome (p-value <0.001), antenatal care practice (p-value <0.001), ethnicity (p-value < 0.001), women’s autonomy (p-value 0.029), wealth quintile (p-value 0.063), and time taken to reach the health institution (p-value 0.001). The regression analysis showed that antenatal care practice (AOR 6.67, 95% CI 2.8-15.93), adverse pregnancy outcome (AOR 0.37 95% CI 0.16-.88), ethnicity (AOR 4.34, 95% CI 1.79-10.5) and time taken to reach the health institution (AOR 2.46, 95% CI 1.1-5.52) were significantly associated with institutional delivery. Mothers who had four or more antenatal care visits, who had not experienced adverse pregnancy outcome in the past, who were from privileged ethnicity group and who could reach the health institution within 60 minutes were more likely to utilize birthing facilities.

**Table 3:** Association of individual, household and health service factors with institutional delivery

**Characteristics**	**Home (n = 83)**	**Institution (n = 157)**	**p-value**	**OR (95% CI)**	**p-value**	**AOR (95% CI)**
Parity						
Multiparous	54	80	Ref.			
Primiparous	29	77	0.036	1.79 (1.03-3.1)	0.664	1.17 (0.58-2.36)
Literacy status (mother)						
Illiterate	37	35	Ref.			
Literate	46	122	<0.001	2.8 (1.58-4.97)	0.476	1.34 (0.6-2.98)
Literacy status (father)						
Illiterate	34	3	Ref.			
Literate	152	51	0.02	3.8 (1.12-12.91)	0.673	0.81 (0.31-2.15)
Occupation (mother)						
Agriculture/Labour	79	139	Ref.			
Business/Service	4	18	0.09	2.56 (0.84-7.82)	0.982	0.98 (0.25-3.82)
Occupation (husband)						
Agriculture/Labour	68	93	Ref.			
Business/Service	15	64	<0.001	3.12 (1.64-5.94)	0.349	1.48 (0.65-3.36)
Wealth quintile						
First quintile (Poor)	24	24	Ref.			
2	13	35	0.023	2.69 (1.15-6.31)	0.229	1.89 (0.67-5.34)
3	15	33	0.063	2.2 (0.96-5.06)	0.936	1.04 (0.37-2.93)
4	15	33	0.063	2.2 (0.96-5.06)	0.832	1.13 (0.37-3.43)
Fifth quintile (least poor)	16	32	0.1	2.0 (0.88-4.56)	0.184	0.48 (0.17-1.41)
Women’s autonomy						
Low	32	48	Ref.			
Medium	32	48	1	1.0 (0.53-1.88)	0.542	0.78 (0.36-1.71)
High	19	61	0.029	2.14 (1.08-4.23)	0.641	1.23 (0.51-3.0)
Ethnicity group						
Underprivileged	74	95	Ref.			
Privileged	9	62	<0.001	5.37 (2.5-11.5)	0.001	4.34 (1.79-10.5)
Experience of adverse pregnancy outcome						
No	56	140				
Yes	27	17	<0.001	0.25 (0.18-0.5)	0.024	0.37 (0.16-0.88)
Time to reach institution						
> 60 minutes	26	20	Ref.			
< 60 minutes	57	137	0.001	3.12 (1.61-0.04)	0.029	2.46 (1.1-5.52)
Antenatal care visits						
< 4 times	33	16	Ref.			
≥4 times	50	141	<0.001	5.82 (2.95-11.46)	<0.001	6.67 (2.8-15.93)

## Discussion

This study was conducted to identify the determinants for the utility of institutional delivery in rural areas of Kavrepalanchowk district of Nepal. Antenatal care service utilization of four or more times was significantly associated with the practice of institutional delivery. Studies in Afghanistan, Nepal and Bangladesh have shown antenatal care to be one of the determining factors for institutional delivery [2,6,11]. It could be inferred that increasing antenatal care coverage in a community increased the probability of institutional delivery. Further, quality antenatal care could play an important role in motivating women of reproductive age to utilize institutional delivery services by gaining the trust of the prospective mothers.

Two studies conducted in Nepal showed that adverse pregnancy outcome was related to increased delivery service utilization [8,11]. Interestingly, adverse pregnancy outcome had an inverse effect on institutional delivery in this study. The observed association could probably be explained by the fact that more adverse pregnancy outcomes occur in home delivery. Mothers who had delivered at home may consider home delivery as normal cultural practice. These mothers may not be aware of the importance of health institution based delivery services. The issue of adverse pregnancy outcome, however, remained elusive, and qualitative studies could help in exploring the reasons in the context of rural Kavrepalanchowk. Mothers from privileged ethnicity were more likely to deliver in health institutions. This might be because privileged ethnic groups were more likely to be financially stable and educated. Research conducted in Nepal and India have shown a significant association between ethnicity and place of delivery [2,8,11,17].

Various studies have shown that the time taken to reach the health institution influenced delivery service utilization [6,11,18,19]. In the current study, the time taken to reach the health institution significantly affected the choice of place of delivery. Accessibility, measured in terms of time to reach health institution, demonstrated that it was critical to ensure the proximity of health services to the communities.

The analysis of perception of health workers’ behaviour during antenatal care was limited to bivariate analysis due to the exclusion of 19 respondents, who did not utilize antenatal care at all. Bivariate analysis showed that the behaviour of health workers during antenatal care was significantly associated (OR 2.89, 95% CI 1.57–5.33) with the utilization of delivery services. This could be because most attitude formation took place during antenatal care, and positive attitudes towards health services affected the utilization levels. This association has been also been observed and explained in a maternal health programme of Nepal [20].

Wealth quintile and women’s autonomy were not significantly associated with delivery service utilization in the study district. However, the role of both wealth quintile and autonomy cannot be ruled out. Studies have shown that higher wealth quintile and better decision making were associated with institutional delivery [2,21].

Although factors such as parity, occupation and education were significantly associated in bivariate analysis, they were not, however, significantly associated in regression analysis. The studies in Pakistan and Nepal showed that lower parity women were more likely to deliver in health facilities and use skilled birth attendants [2,5,8,11,22]. However, the Botswana study showed that lower parity was associated with home delivery [10]. In the current study, education and occupation were also not significantly associated with place of delivery, although other studies have established an association [5,8,11,22-24].

The study had some notable limitations. The study may be affected by study design bias, social-desirability bias, recall bias and/or purposive selection of study area. Cross-sectional studies generally identify association but have difficulty in establishing temporal relationship [25,26]. Mothers may have been more likely to report socially desirable behaviours instead of actual practices related to maternal health due to the use of local enumerators. There was a possibility of under-reporting of adverse pregnancy outcome because of its sensitive nature.

## Conclusion

Mothers with antenatal care check-up of four or more times, from privileged ethnicity, and with travel time less than 60 minutes to reach the health institution were more likely to practice institutional delivery. Experience of adverse pregnancy outcome was inversely associated with institutional delivery. Utilization of institutional delivery was also affected by perception of mothers towards the behaviour of health workers. Universal coverage of antenatal care may ensure the increased utilization of health institution for delivery. Programs aiming to promote utilization of maternal health services should focus on regions with a high proportion of disadvantaged ethnic groups. There is need to explore why mothers who had experienced adverse pregnancy outcome were less likely to attend health facilities for delivery services in Kavrepalanchowk district.

## Abbreviations

FCHV: Female community health volunteers; VDC: Village development committee; PCA: Principle component analysis.

## Competing interests

The authors declare that they have no competing interests.

## Authors’ contributions

SRS was responsible for the design, analysis and preparation of the article; AKP, BMD and SS supported in design and analysis. All authors read and approved the final manuscript.

## Pre-publication history

The pre-publication history for this paper can be accessed here:

http://www.biomedcentral.com/1471-2458/14/306/prepub
